# Refractory Epstein-Barr Virus-Negative Diffuse Large B-Cell Lymphoma Post-Transplant Lymphoproliferative Disorder in a Renal Transplant Patient Treated With Bispecific Antibody Therapy

**DOI:** 10.14740/jmc5186

**Published:** 2025-10-10

**Authors:** Austin Frisch, Bobby Se, Eric Martin, Haresh Mani, Stephen Medlin, Zartash Gul

**Affiliations:** a Department of Internal Medicine, Inova Fairfax Medical Center, Falls Church, VA, USA; b Department of Pathology, Inova Fairfax Medical Center, Falls Church, VA, USA; c Inova Schar Cancer Center, Inova Fairfax Medical Center, Falls Church, VA, USA

**Keywords:** DLBCL, PTLD, Transplant, Bispecific antibody, Lymphoma

## Abstract

Post-transplant lymphoproliferative disorder (PTLD) is a rare disorder that can occur in both stem cell and organ transplant recipients. Most cases of PTLD arise from Epstein-Barr virus (EBV)-induced oncogenesis but it can be EBV-negative. The treatment algorithm involves reduction in immunosuppression combined with anti-CD20 antibody treatment followed by chemotherapy. Despite this, mortality is still high, and many patients have refractory disease. Refractory PTLD is challenging to treat, and the data on treatment are limited. Both stem cell transplant and chimeric antigen receptor T-cell (CAR-T) therapies have been described in case reports and small reviews, but success has been limited. Hence, there is a need for novel therapies and clinical trials to formulate better treatment plans. Here, we present a complex case of refractory EBV-negative PTLD that progressed on third-line treatment. Given the patient’s limited options, glofitamab, a bispecific antibody therapy, was initiated in attempt to bridge to CAR-T therapy. Unfortunately, the patient encountered multiple hospitalizations and eventually elected for hospice care not long after initiation of this fourth-line regimen began. Herein, we detail the diagnosis and treatment options for EBV-negative refractory PTLD with an in-depth literature review that highlights bispecific antibodies as a potential novel therapy.

## Introduction

Post-transplant lymphoproliferative disorder (PTLD) is a rare malignancy that can occur after organ transplantation. PTLD incidence varies depending on which organ is transplanted and patients who receive multi-organ transplants are at the highest risk. Kidney transplants tend to have the lowest incidence of PTLD cases with 1.58 per 1,000 persons/year and a 25-year cumulative PTLD incidence of roughly 3.3% [1, 2]. Immunosuppression (IS) and chronic viral infection, particularly Epstein-Barr virus (EBV), are the major risk factors for PTLD and the highest risk occurs within the first year of transplant [3]. This disease can present locally or advanced with the most common locations being gastrointestinal or lymph nodes and the most common symptoms being fever and weight loss. Most PTLD cases are EBV-positive (60-80%), while EBV-negative cases are extremely rare [4]. Treatment of PTLD includes cytotoxic chemotherapy, targeted antibody therapy, and reduction of IS. However, it is difficult to determine efficacy of these therapies leading to poor outcomes (overall survival 60%) due to the rarity and heterogeneity of this disease [5].

The standard of care for PTLD involves the reduction of IS by about 50% and treatment with an anti-CD20 monoclonal antibody such as rituximab or obinutuzumab. Chemotherapy regimens such as rituximab, cyclophosphamide, doxorubicin, vincristine, and prednisone (R-CHOP) can be used when PTLD are refractory to first-line therapies. Beyond this, exciting new research and clinical trials are underway testing the efficacy of bispecific antibody therapies and antibody drug conjugates in PTLD.

Bispecific antibodies, such as epcoritamab or glofitamab, bind to CD20 on B cells and CD3 on T cells to facilitate their interaction with PTLD cells and trigger T-cell destruction of the malignancy [6]. Bispecific antibodies are an emerging treatment option for PTLD; however, they are still in clinical trials. Here, we present a rare case of EBV-negative PTLD following a renal transplant treated with bispecific antibody that progressed despite multiple lines of treatment.

## Case Report

### Investigations

**Figure 1:**
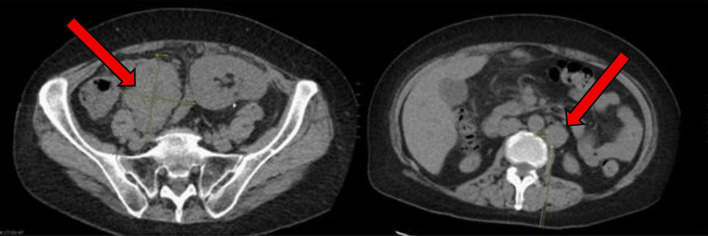
At time of initial presentation, CT abdomen and pelvis showing increase in size of the abdominal and pelvic lymph nodes (arrows) suspicious for post-transplant lymphoproliferative disorder. CT: computed tomography.

A 57-year-old woman with a history of end-stage renal disease (ESRD) due to IgA nephropathy underwent renal transplant from her sibling. She was placed on standard immunosuppressive therapy with tacrolimus 1 mg twice daily, mycophenolate mofetil 750 mg twice daily, and prednisone 5 mg daily. Nearly 2 years after transplant, she began to develop progressive abdominal discomfort, early satiety, and unintentional weight loss. These symptoms continued to progress over the next 6 months, thus leading to her presentation 30 months after transplant. On the initial examination, she had no overt lymphadenopathy or fever. Laboratory tests showed a white blood cell count of 11.07 × 10^3^/µL, hemoglobin of 10.3 g/dL, and platelet count of 568 × 10^3^/µL. Kidney function remained stable with a creatinine of 0.9 mg/dL, sodium of 139 mEq/L, and potassium of 4.1 mEq/L. Liver function testing was notable for an aspartate aminotransferase (AST) of 50 U/L, alkaline phosphatase (ALP) of 275 U/L, and albumin of 3.4 g/dL. The patient underwent a computed tomography (CT) scan of the abdomen and pelvis, which revealed multiple enlarged mesenteric lymph nodes, liver lesions, and thickening of the terminal ileum (Fig. 1). Notably, the liver mass was heterogeneous and fludeoxyglucose-18 (FDG)-avid on positron emission tomography (PET) scan. Given the concern for malignancy, a retroperitoneal lymph node biopsy was performed.

### Diagnosis

**Figure 2:**
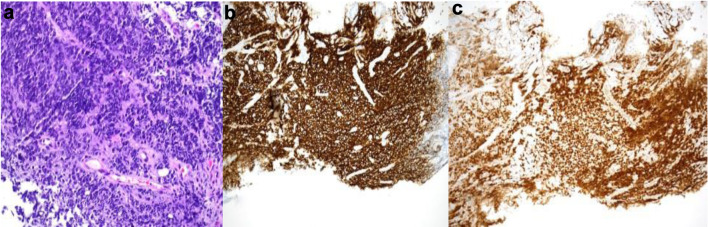
Gastric biopsy showing sheets of small round blue tumor cells (a, H&E, × 200) that stained diffusely positive with B-cell markers CD20 (b, CD20 immunohistochemistry, × 100) and PAX5 (c, PAX5 immunohistochemistry, × 100). H&E: hematoxylin and eosin.

The pathology results confirmed the diagnosis of diffuse large B-cell lymphoma (DLBCL), a subtype consistent with PTLD. Figure 2 shows the biopsy and stains confirming the diagnosis. Stains are positive for CD20 and PAX5 (Fig. 2), but also positive for CD10, CD79a, and Ki-67. Immunohistochemical (IHC) analysis did not detect EBV via fluorescent *in situ* hybridization (FISH), and the lymphoma was classified as EBV-negative DLBCL. This was further supported by negative BCL6, MYC, and BCL2 rearrangements based on FISH analysis, suggesting a germinal center B-cell origin.

### Treatment and outcome

Almost 30 months after her kidney transplant, she started the first cycle of R-CHOP. Despite experiencing mild neutropenia and nausea as side effects, the patient tolerated the initial treatment well. A CT scan after the second cycle showed partial regression of the lymphadenopathy and resolution of the liver lesions.

**Figure 3:**
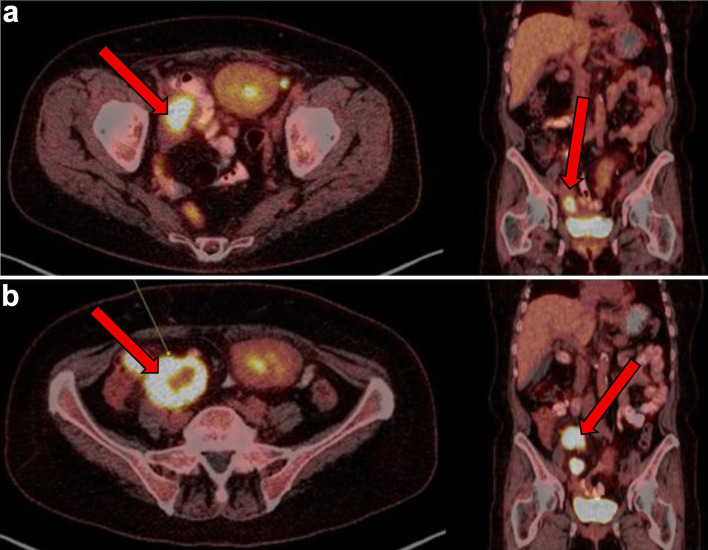
(a) PET showing metabolic activity of right sided pelvic lymph nodes/nodal masses (arrows) at 3 months from initial presentation. (b) PET conducted 4 months after initial presentation with diffuse terminal ileum/ascending colon thickened wall uptake and increase in size and metabolic activity of right-sided pelvic lymph nodes/nodal masses (arrows), consistent with persistent lymphoma. PET: positron emission tomography.

Following six cycles of R-CHOP however, 4 months after her EBV-negative DLBCL diagnosis, the patient’s disease was noted to have progressed. New lesions were identified on a follow-up PET scan, including right pelvic lymph nodes, ileal thickening, and ascending colon lesions, which were concerning for intestinal involvement (Fig. 3). She subsequently had a biopsy of this right pelvic lymph node which showed focal residual DLBCL, germinal center type with extensive necrosis. Biopsy showed that large atypical lymphoid cells are positive for BCL6, CD10, CD20, CD79a, PAX5, and Ki-67 (over 90%) stains.

Initiation of rituximab and polatuzumab vedotin was delayed by nearly 2 months due to several hospitalizations after this biopsy. And, shortly following the initiation of this therapy line, she was hospitalized in the intensive care unit for septic shock. A repeat CT of abdomen and pelvis showed persistent disease and pathology from an upper and lower endoscopy revealed a gastric ulcer with B-cell lymphoma and cytomegalovirus colitis. This prompted a multidisciplinary evaluation with infectious disease, transplant surgery, and malignant hematology, with the recommendation to proceed with chemotherapy to reduce lymphoma burden while maintaining prophylactic antimicrobials including valganciclovir. She was started on rituximab, gemcitabine, and oxaliplatin (R-GemOx) nearly 4 months after completion of R-CHOP. She received four cycles of R-GemOx.

In the next 4 months, the patient reported worsening abdominal pain, thus prompting repeat abdominal CT imaging, which unfortunately revealed further progression of disease. She was then referred to bone marrow transplant hematology for consideration of advanced cellular therapies. Now, almost 12 months out from initial EBV-negative DCLBCL diagnosis, it was recommended to start this patient on obinutuzumab and glofitamab as a bridge to chimeric antibody replacement therapy. She was admitted to the hospital after being seen for her first infusion of obinutuzumab and glofitamab for intravenous hydration and monitoring of cytokine release syndrome (CRS) then subsequently discharged. The patient did not develop CRS.

**Figure 4:**
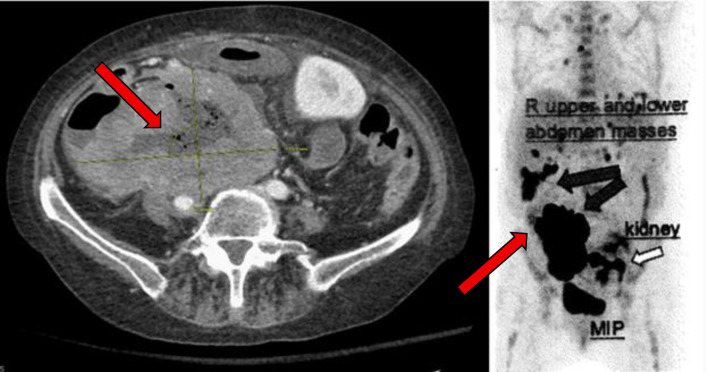
PET scan with diffuse disease progression (arrows) 14 months after initial presentation. PET: positron emission tomography.

A month later, polatuzumab vedotin was added to her glofitamab and obinutuzumab as part of her treatment plan. Shortly thereafter, she was hospitalized for *C. difficile* colitis and abdominal pain. CT imaging revealed new ascites and further disease progression, including increased intra-abdominal tumor burden with measurements of a tumor measuring 6.5 × 9.1 cm a month prior, now progressing to 7.9 × 11.9 cm. An additional PET CT was performed a month later, which showed further disease progression (Fig. 4). Therefore, it was recommended to start the patient on glofitamab and loncastuximab tesirine-lpyl based on the LOTIS-7 trial.

**Figure 5:**
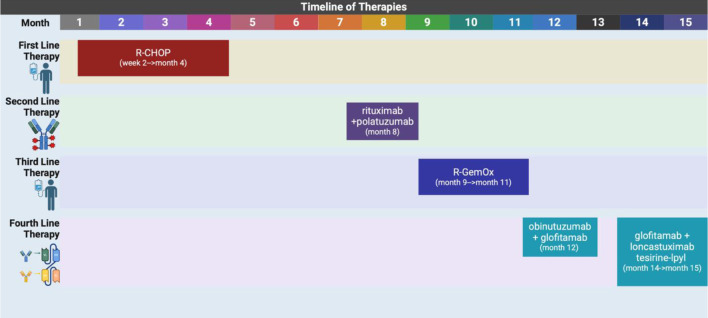
A timeline showing the different lines of therapies used to treat this case. R-CHOP: rituximab, cyclophosphamide, doxorubicin, vincristine, prednisone; R-GemOx: rituximab, gemcitabine, oxaliplatin. Created in BioRender by A. Frisch (2025). https://BioRender.com/9hy92es

She received her first dose of glofitamab and loncastuximab tesirine-lpyl nearly 15 months after her initial diagnosis. She was admitted to the hospital a week later due to abdominal pain, fever, and chills with imaging at the time that did indicate her disease had progressed. However, after having extensive conversations with the medical team and patient’s family, the patient expressed desire to stop aggressive treatment and elected to transition to hospice care. At that time, she reported symptomatic relief with an effective pain regimen and was at peace with her decision to forgo further treatment. The palliative care team was involved in supporting the patient’s decision. Hospice services were continued, and she remained under close observation with continued emotional and pain management support. Table 1 describes in detail this patient’s complicated clinical course and treatment history. Figure 5 is a visual timeline of the therapies used to treat this case.

**Table 1:** Patient’s Complicated Clinical Course and Treatment History

Timeline	Clinical events and findings	Management	Outcome
30 M prior to initial presentation	Deceased donor kidney transplant for ESRD (hypertensive nephrosclerosis).	Immunosuppression with tacrolimus, mycophenolate, prednisone.	Stable graft function post-transplant.
6 M prior to initial presentation	Progressive abdominal discomfort, early satiety, unintentional weight loss.	Symptom monitoring.	Worsening symptoms over months.
Initial presentation, “day 0”	Abdominal pain and fatigue. CT abdomen and pelvis showing increase in size of the abdominal and pelvic lymph nodes suspicious for post-transplant lymphoproliferative disorder (Fig. 2).	Retroperitoneal lymph node biopsy.	Diagnosed with EBV-negative DLBCL, germinal center B-cell subtype PTLD.
2 W after presentation	Initiated first cycle of R-CHOP.	Rituximab, cyclophosphamide, doxorubicin, vincristine, prednisone.	Mild neutropenia, partial radiographic response after two cycles.
4 M after presentation	Disease progression post six cycles of R-CHOP. PET: new pelvic nodes, bowel lesions (Fig. 3).	Biopsy of right pelvic node: residual DLBCL with necrosis, high Ki-67, GC phenotype.	Confirmed refractory disease.
8 M after presentation	Started on rituximab + polatuzumab vedotin (delayed due to hospitalizations).	Chemotherapy initiated.	Admitted to ICU with septic shock post-cycle.
8 - 9 M after presentation	EGD/colonoscopy: gastric ulcer (DLBCL), CMV colitis.	Multidisciplinary discussion; suppressive antibiotics.	Decision to continue therapy.
9 - 11 M after presentation	Four cycles of R-GemOx.	Tolerated treatment initially.	CT in October: disease progression with worsening abdominal pain.
12 M after presentation	Bridging therapy initiated: obinutuzumab + glofitamab for CAR T-cell consideration.	Admitted for CRS monitoring after first dose.	Discharged, tolerated initial treatment.
13 - 14 M after presentation	Polatuzumab added. Recurrent admissions for GI toxicity (*C. difficile* colitis, pain).	Supportive care, pain control.	CT: tumor progression (up to 11.9 cm mass), new ascites.
14 - 15 M after presentation	PET scan confirms disease progression (Fig. 4).	Started glofitamab + loncastuximab tesirine-lpyl (LOTIS-7 trial).	Admitted soon after first dose with pain, fevers.
15 M after presentation	Emotional distress, ongoing symptoms. Imaging: no new disease.	Goals-of-care discussion. Transitioned to hospice care.	Patient chose to stop treatment.

“M” represents months in the timeline. “W” represents weeks in the timeline. The table begins with the patient’s kidney transplant and early symptoms then arriving “day 0” with our inpatient team’s first encounter. The following rows detail her treatment course and disease progression. CAR: chimeric antigen receptor; CMV: cytomegalovirus; CRS: cytokine release syndrome; CT: computed tomography; DLBCL: diffuse large B-cell lymphoma; EBV: Epstein-Barr virus; EGD: esophagogastroduodenoscopy; ESRD: end-stage renal disease; GI: gastrointestinal; ICU: intensive care unit; PET: positron emission tomography; PTLD: post-transplant lymphoproliferative disorder; R-CHOP: rituximab, cyclophosphamide, doxorubicin, vincristine, prednisone; R-GemOx: rituximab, gemcitabine, oxaliplatin.

## Discussion

EBV-negative PTLD is a rare disease with poor prognosis. To date, there has been no published case on using bispecific antibody therapy to treat refractory EBV-negative DLBCL PTLD. Most scholars assume that the driving force behind this disease is intense IS and transplant-related chronic antigen stimulation rather than viral oncogenesis [7, 8]. Further studies have highlighted the similarities between gene expression and molecular signatures in EBV-negative DLBCL PTLD and DLBCL in immune-competent patients which are distinct from EBV-positive DLBCL PTLD [8, 9]. Pointing towards a non-viral mediated etiology that is more like classical DLBCL pathogenesis. Reduction in IS regimen is a first-line therapy for PTLD but has shown mixed results. A 2008 study with only 16 PTLD patients that reduced tacrolimus or cyclosporine by 50% reported only a 12% partial response rate and no patients were put into remission all while graft rejection approached 40% [10]. A larger 2011 study with 67 PTLD patients had a complete response of 37%, but graft rejection was still high at 32% [11]. For EBV-negative PTLD, systemic anti-CD20 antibody therapy is indicated along with reduction in IS. A 2010 multicenter analysis of 80 PTLD patients showed improved progression-free survival and overall survival with the addition of rituximab to PTLD patients who underwent IS reduction [12]. Further advancements in PTLD treatment were made with the data from the landmark trial PTLD-1. This study demonstrated that 4 weeks of monotherapy with rituximab followed by four 21-day cycles of cyclophosphamide, doxorubicin, vincristine, and prednisone (CHOP) resulted in a 90% of patients having complete or partial response with a mean overall survival of 6.6 years [13]. A retrospective study done in 2010 compared R-CHOP with rituximab, prednisone, etoposide, vincristine, cyclophosphamide, doxorubicin (R-EPOCH) in PTLD patients and showed a 2-year overall survival for the R-EPOCH group of 76.1% vs. 41.7% for the R-CHOP group, proving R-EPOCH as a viable treatment strategy [14, 15].

Our patient underwent standard therapy with reduction in IS followed by R-CHOP but, unfortunately, had aggressive refractory disease even after another round of rituximab with polatuzumab vedotin. A combination of gemcitabine and oxaliplatin (GemOx) has been shown to have almost a 50% overall response rate and a 2-year overall survival of 44% in relapsed DLBCL patients proving to be an option for salvage therapy [16]. Beyond the trial of R-GemOx, our patient had very few options, and data supporting other therapies were scarce if not unavailable. Because of such limited data on refractory EBV-negative DLBCL PTLD, standard practice has become following the algorithm for refractory/relapsed DLBCL in non-PTLD patients. Salvage therapy followed by autologous stem cell transplant (ASCT) is not routinely used in PTLD patients and in a large review of over 3,600 cases, only 9.7% had ASCT [17]. Chimeric antigen receptor T-cell (CAR-T) therapy in DLBCL PTLD cases is not common and evidence is limited [18-20]. A retrospective study that included 22 relapsed refractory solid organ PTLD patients who underwent CAR-T therapy showed an overall response rate and complete remission of 64% and 55% respectively with 18/22 patients experiencing CRS [20]. For our patient, since she was failing conventional salvage therapy, the decision was made to use bispecific antibody therapy according to the LOTIS-7 trial as a bridge to CAR-T therapy. The LOTIS-7 trial is an ongoing phase I trial testing the safety, efficacy, and tolerability of loncastuximab tesirine (a CD-19 antibody) with glofitamab (a bispecific antibody) in patients with relapsed or refractory DLBCL (NCT04970901). Should she relapse with CAR-T therapy, then a ASCT would be considered. However, given the state of her health and functional status, the patient and medical team acknowledged that if she continues to worsen with continued hospitalizations, then these future therapies cannot be offered.

There are currently no data on the safety or efficacy of bispecific antibody use in PTLD. First approved in 2022 for refractory B-cell lymphoma, this therapy can come with numerous side effects including CRS, neurotoxicity, and cytopenia [21, 22]. In a phase II study testing glofitamab in 155 refractory DLBCL patients, 39% of patients had a complete response and the 12-month progression-free survival was 37% despite almost two-thirds of patients having a grade 3 or higher adverse event [21]. Currently, epcoritamab is being studied in a clinical trial in patients with relapsed or refractory B-cell PTLD (NCT0245886) and blinatumamab, another bispecific antibody, has also been shown to be effective in PTLD patients [23]. Despite the limited data in PTLD patients, using bispecific antibodies was the next logical step after our patient progressed on three lines of therapy.

In future cases of EBV-negative DLBCL PTLD in kidney transplant recipients, we recommend an early risk stratification approach that prioritizes immune-based therapies over traditional cytotoxic chemotherapy. This strategy may help mitigate T-cell exhaustion and reduce the emergence of resistant malignant clones, which are common challenges in heavily pretreated patients. Early introduction of targeted antibody therapies while maintaining IS in the disease course could potentially improve outcomes and reduce toxicity. Another important consideration is the origin of lymphoma cells - whether they are donor- or recipient-derived. Increasing reports of donor-derived hematologic malignancies following kidney transplantation highlight the need for thorough investigation. Although further sampling is not possible in this deceased patient, we recognize the importance of incorporating donor-versus-recipient origin analysis into standard diagnostic workup, especially in cases of atypical or refractory PTLD.

### Conclusion

This case underscores the complexities of managing EBV-negative DLBCL PTLD in kidney transplant patients. We present the first reported use of bispecific antibody therapy following the failure of multiple treatment regimens. The patient’s multiple relapses and hospitalizations highlight the importance for a multidisciplinary approach within a high-resource setting. Personalized treatment planning is crucial for successful outcomes, and early molecular profiling along with EBV testing can help guide urgent PTLD management. Novel therapies such as glofitamab combined with loncastuximab tesirine offer a promising option for refractory disease in patients who are unfit for ASCT or CAR-T therapy, or who have not responded to salvage therapy. Further studies are needed to confirm the efficacy of bispecific antibody treatments in refractory DLBCL PTLD, and clinical trials are already underway.

### Learning points

EBV-negative DLBCL PTLD is an extremely rare disease with poor outcomes.

The data are limited for treatment options in refractory PTLD cases, leading physicians to use guidelines for refractory DLBCL in non-PTLD patients.

Using bispecific antibodies as treatment for refractory DLBCL PTLD has never been published as a case report until now and continues the discussion around this new realm of treatment options for this complex disease.

More research is needed to further characterize the standard of treatment for refractory EBV-negative DLBCL PTLD and novel therapies, such as bispecific antibodies, are promising.

## Data Availability

The authors declare that the data supporting the findings of this study are available within the article.
